# Analysis of ecological thresholds in a temperate forest undergoing dieback

**DOI:** 10.1371/journal.pone.0189578

**Published:** 2017-12-14

**Authors:** Philip Martin, Adrian C. Newton, Elena Cantarello, Paul M. Evans

**Affiliations:** Faculty of Science and Technology, Bournemouth University, Talbot Campus, Poole, United Kingdom; Technical University in Zvolen, SLOVAKIA

## Abstract

Positive feedbacks in drivers of degradation can cause threshold responses in natural ecosystems. Though threshold responses have received much attention in studies of aquatic ecosystems, they have been neglected in terrestrial systems, such as forests, where the long time-scales required for monitoring have impeded research. In this study we explored the role of positive feedbacks in a temperate forest that has been monitored for 50 years and is undergoing dieback, largely as a result of death of the canopy dominant species (*Fagus sylvatica*, beech). Statistical analyses showed strong non-linear losses in basal area for some plots, while others showed relatively gradual change. Beech seedling density was positively related to canopy openness, but a similar relationship was not observed for saplings, suggesting a feedback whereby mortality in areas with high canopy openness was elevated. We combined this observation with empirical data on size- and growth-mediated mortality of trees to produce an individual-based model of forest dynamics. We used this model to simulate changes in the structure of the forest over 100 years under scenarios with different juvenile and mature mortality probabilities, as well as a positive feedback between seedling and mature tree mortality. This model produced declines in forest basal area when critical juvenile and mature mortality probabilities were exceeded. Feedbacks in juvenile mortality caused a greater reduction in basal area relative to scenarios with no feedback. Non-linear, concave declines of basal area occurred only when mature tree mortality was 3–5 times higher than rates observed in the field. Our results indicate that the longevity of trees may help to buffer forests against environmental change and that the maintenance of old, large trees may aid the resilience of forest stands. In addition, our work suggests that dieback of forests may be avoidable providing pressures on mature and juvenile trees do not pass critical thresholds.

## Introduction

An ecological threshold is the point at which a relatively small change results in a rapid, non-linear response, in ecosystem properties [1]. Such thresholds have recently attracted attention owing to the concern that the environmental impacts of human activities may lead to rapid ecological change resulting from relatively minor changes in human pressures [2–4]. For example, a small increase in precipitation or temperature could lead to major changes in regional or global climate, resulting in ecosystems transitioning from one state to another [5]. While the conceptual framework underlying ecological thresholds has been shown to be applicable across a range of ecosystems, the mechanisms responsible for rapid shifts in ecosystems are often unclear [6].

Ecological thresholds are thought to occur when a controlling variable shifts from a negative feedback to a positive feedback [7]. In forest ecosystems, disturbances such as tree cutting, fire, or drought can interact either with each other or with other pressures, resulting in positive feedbacks that can drive the system towards a different state [1]. However, these feedbacks can be difficult to detect because drivers may operate over very different spatial and temporal scales [8]. In forest ecosystems, feedbacks of particular concern are those between local disturbances (e.g. fire, pests, drought, browsing/grazing or deforestation) and climatic changes [8]. For example, it is thought that logging and deforestation in tropical forests combined with increased drought and frequency of fires may result in a shift to savannah-like vegetation structure [9,10]. Similarly, large-scale disturbances in Mediterranean forests can lead to reduced seedling recruitment and invasion by grasses and shrubs, which result in increased fire frequency and further suppression of tree cover [11].

Empirical evidence for ecological thresholds is accumulating, but there are relatively few field studies of this phenomenon, particularly in terrestrial ecosystems [12–15]. In forests, thresholds have been identified in relation to grazing pressure, landscape fragmentation, patch size, and connectivity [16,17]. Increasingly, however, researchers are voicing concerns regarding the incidence of large-scale disturbance events affecting forests, including increasing temperatures, drought, insects and pathogen outbreaks, and uncharacteristically severe wildfire [18–20]. Such factors may interact with other anthropogenic stressors, such as atmospheric pollution and invasive species, to cause extensive forest dieback [21,22] which can result in shifts to relatively treeless, non-forest states [8]. Over the past decade research has suggested that such shifts may occur in both tropical [10,23] and boreal regions [24] as a result of changes in climate and disturbance regimes. Forests may be particularly vulnerable to rapid environmental changes because trees are relatively long-lived, immobile organisms that consequently find it difficult to adapt to new environmental conditions [25,26]. Any shift to non-forest states would cause loss of forest biodiversity as well dramatic changes in the provision of ecosystem services [27], which could have major economic implications [28]. Despite these concerns, relatively little is known about the mechanisms by which forest ecosystems might transition to relatively treeless states, and whether such transitions are characterised by ecological thresholds [8].

Here we examine the mechanisms underlying ecological thresholds in a temperate forest ecosystem that has undergone partial dieback in recent decades. At this site, data have been collected repeatedly over a period of 50 years from 1964–2014 [29–32]. Analysis of this long-term data set has indicated that basal area (BA) in the forest has declined by 33% over this period, and juvenile tree densities have been reduced by approximately 70% [31]. Evidence suggests that an increasing incidence of drought has interacted with attack by pathogenic *Phytophthora* fungi to cause tree mortality, particularly of large individuals [31]. Previous work suggests a number of different ecological thresholds were associated with this dieback, such as non-linear changes in tree species composition, grass cover, and ground flora species richness in relation to changes in BA (31, 32). In the current investigation, we build upon this previous research by examining whether BA decline is itself characterised by an ecological threshold. We then examine the mechanisms underlying stand dieback. Specifically, we hypothesize that a positive feedback between mortality of mature trees (caused by drought and pathogen attack) and of juveniles (caused by herbivory) may account for a threshold response of BA over time, leading to a transition to a forest with a more open canopy, and fewer trees. Using statistical models we investigate the factors influencing tree mortality and recruitment, both of which include potential feedback mechanisms. We then use these statistical models to inform an individual based model to test the effects of hypothesized feedbacks on forest structure.

## Materials and methods

This study was conducted in Denny Wood in the New Forest National Park, Southern England (Lat: 50.89° N Long: -1.54°). Woodland vegetation at the site was dominated by old-growth beech (*Fagus sylvatica*) with frequent pedunculate oak (*Quercus robur*) and birch (*Betula pendula*, *B*. *pubescens*), and an understory of holly (*Ilex aquifolium*). In open areas the ground vegetation was mostly comprised of *Agrostis*-dominated grassland or bracken (*Pteridium aquilinum*). Large populations of deer, ponies and cattle in the New Forest cause high herbivore pressure [33], which Denny Wood has experienced since at least the 1960’s [30]. Since the early 1980’s the site has undergone significant dieback of beech trees [31]. The site has not been subject to direct human disturbance for at least 100 years [30]. In addition to the data collected at Denny Wood we collected data on seedling and sapling densities from plots established along gradients of forest dieback carried out at 12 sites across the New Forest [32], hereafter referred to as gradient sites.

Measurements at Denny Wood were conducted in a 20 m-wide, 1 km long transect. The transect was subdivided into 45 contiguous 20 x 20 m (0.04 ha) subplots and surveyed in 1964, 1984, 1988, 1996 and 2014 (as described in 31–33). In each survey, the location and species of all woody stems >1.3 m in height was recorded, their diameter at breast height (DBH, at 1.3 m) measured, and their status assessed as either alive or dead. Each stem >1.3 m height was given a unique ID number to allow individual trees to be tracked between surveys. Stems <10 cm DBH were classified as saplings and those >10 cm DBH as mature trees. For the gradient sites, five 20 x 20 m survey plots were established in each site along a gradient of woodland dieback, using BA as a measure of forest structure. In each case, beech was the dominant canopy tree species. Plots were situated to provide values of 100%, 75%, 50%, 25% and 0% BA, with 100% representing a relatively intact forest stand and 0% indicating complete death of all canopy trees.

In 2014 we collected data on seedling density, canopy openness, and soil characteristics in Denny Wood and the 12 gradient sites. The density of tree seedlings of all species present in 10 x 10 m plots located in the centre of the 20 x 20 m plots was recorded. Canopy openness of subplots was assessed using a concave spherical densiometer in all four corners and the centre of 20 x 20 m plots, and the mean calculated for each subplot. Soil type was assessed by collecting three soil samples in each 20 x 20 m subplot using a 5 cm diameter soil corer. The first 20 cm of the mineral layer was retained. Soil samples were analysed to quantify particle size distribution, allowing the percentage content of clay, silt and sand to be determined.

### Subplot dynamics

To assess the dynamics of subplots in Denny Wood we analysed the temporal change of BA in plots between 1964 and 2014. To estimate the changes in BA of plots with differing dynamics we divided subplots into those that declined in BA by >25% between 1964 and 2014 and those that did not. We considered plots that had declined by >25% to represent those where substantial dieback or ‘collapse’ had occurred, while others were relatively stable. We then analysed the changes in BA separately using subplot number as a random effect to account for repeated sampling. Plots were split into these two categories to communicate the different dynamics seen across the site.

### Sapling and mature tree mortality

Previous work at Denny Wood has shown that since the 1960’s sapling density has declined [31]. To determine the extent to which this was attributable to growth of individuals into mature trees or to sapling mortality, we tracked the fate of each sapling recorded. For each census period we calculated the annual mortality rate *m* modified from [34] as:

$$m\;=\;1-{\left(\frac{N_{1}-G_{1}}{N_{0}}\right)}^{1/t}$$

where *N*_0_ is the number of saplings in the first survey, *N*_1_ is the number of stems at the second survey, and *G*_1_ is the number of stems that have increased to >10 cm DBH between the first and second survey. To calculate the annual percentage of saplings that grew to >10 cm DBH we used the same equation but replaced *G*_1_ with the number of saplings that died during the census period. To assess possible mechanisms limiting beech recruitment we analysed the relationship between the density of seedlings and saplings, and canopy openness at both Denny Wood and the gradient sites using generalised linear mixed models.

To investigate potential causes of sapling and mature tree mortality, we first assessed if mortality of saplings and mature trees could be explained by self-thinning, a process of intra-specific competition in which stem density decreases as total biomass increases [35]. Previous studies in Denny Wood had suggested that self-thinning was a significant cause of mature tree mortality [30]. To investigate this relationship we used a linear mixed model to relate stem density to BA at the scale of subplots, with subplot number as a random effect. We used BA as a proxy for biomass to test for self-thinning, following [35] and [36].

Following this we investigated the effects of different variables on beech tree mortality. These analyses included trees both classified as saplings and as mature trees. Here, a tree was considered to have died when it had either been recorded as dead or if it was recorded during one census but not at the subsequent census. We selected three non-overlapping census periods for analysis: 1984–1988, 1988–1996 and 1996–2014 (mean census period 10 ± 5.9 years). Statistical models of individual tree mortality were developed using logistic mixed effects models, which describe the probability of a tree dying in a given period of time. To correct for the variation in census interval we used a complementary log-log link with an offset equal to the census interval, so that predictions from models were equivalent to the annual probability of mortality [37]. Subplot ID number was used as a random effect to account for repeated sampling of the same plots [37]. Goodness of fit was tested using le Cessie-van Houwelingen-Copas-Hosmer tests, with P ≤ 0.05 indicating that models are a poor fit [38].

We developed tree mortality models in a four-step process similar to that of Chao et al. [39]. In step one we prepared predictors classified into five groups (i) tree size, (ii) tree growth, (iii) proximity to dead trees, (iv) soil type, and (v) plot stem density. Variables relating to tree size, growth, and plot stem density represent measurements calculated prior to death (for further details see the supplementary materials). All model variables were standardised using the methods of Schielzeth [40] by subtracting the mean of the variable and dividing by its standard deviation. This allows coefficients to be interpreted as effect sizes, reduces collinearity between variables and improves model convergence [40]. In step two we selected the best predictor for each group by choosing the models that had the lowest AICc [39,41]. This step reduces intercorrelation of variables, which can lead to difficulty in interpreting effects [39]. In step three a full multivariate model was developed using these selected variables using additive terms only. In step four model averaging was used to produce parameter estimates for models with a ΔAICc≤7. All analyses were conducted using R 3.4.0 [42] with generalised linear mixed models performed using the lme4 package [43] and multimodel averaging using the MuMIn package [44].

### Individual based model

To test our hypothesis of a positive feedback of the death of large, old trees resulting in relatively open forest areas that were preferentially grazed by livestock that impaired seedling recruitment we developed an individual based model, built using Netlogo [45]. For a detailed description of the model see the supplementary materials. The model simulates recruitment, death and growth in a four hectare forest stand where all individuals represent the canopy dominant species in Denny Wood, beech (*Fagus sylvatica*). The model contains sub-models to simulate tree reproduction, growth, mortality as a function of tree size and growth-rate, size-asymmetric density dependant mortality, and occurrence of feedbacks in juvenile mortality. Parameters used in the model were taken from the empirical data presented in this study or from relevant scientific literature (S1 Table).

We initiated the model so that size structure, stem density, and BA were similar to values observed in Denny Wood in 2014 and ran this model for 100 time steps, for 242 different scenarios. Each scenario consisted of different values representing the probability of an individual seedling or sapling/mature tree dying in a single year, thus simulating the effect of different intensities of grazing pressure in the forest. Sapling/mature tree mortality probability increased as the size of the trees increased, following results of our statistical modelling. However, we varied the base mortality probability of sapling/mature trees by altering the value for the intercept in the regression equation obtained from the statistical analyses. This allowed us to simulate size-dependant mortality as well as the potential effects of changes in mortality probability for trees of all sizes. We varied the annual probability of mortality for seedlings between 0–1 and for sapling/mature trees the intercept varied between 0–0.05. In addition, we used a variable to simulate a feedback in seedling mortality, as a result of increased grazing intensity, in which seedlings died if they occurred in forest gaps. Forest gaps were classified as areas with <50% canopy cover (see supplementary materials for details of how this was calculated). The feedback variable was switched on and off for all combinations of seedling and sapling/mature tree mortality probabilities, allowing us to assess the potential impact of the feedback on forest structure. At each time step BA, mature tree stem density, and canopy cover were recorded. Each model run had 100 iterations and median values were used to summarise model results.

## Results

**Fig 1:**
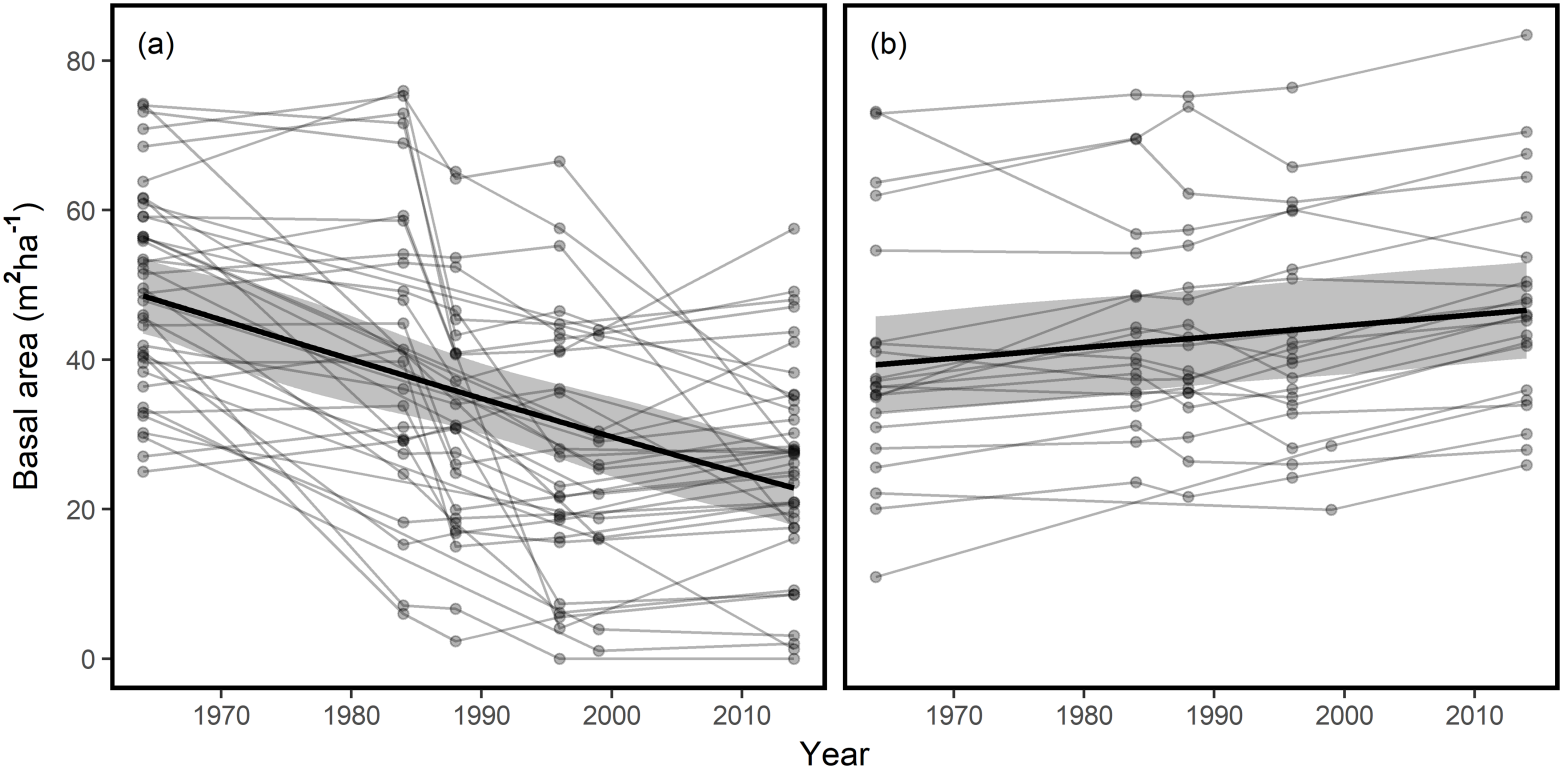
Changes in subplot basal area. Changes in basal area for plots that (a) declined in basal area by >25% or (b) showed declines of <25% or increased in basal area. Solid black lines represent predictions from linear mixed models, with grey area representing 95% confidence intervals for these predictions. Points represent individual subplots and grey lines represent the trajectory of these subplots.

To visualise the trajectories of BA change, subplots were divided into two groups, where BA decline was >25% (Fig 1a) and where BA decline was <25% or was stable (Fig 1b) over the census period. In those subplots where BA declined, non-linear trajectories were observed in a number of subplots (Fig 1a). Particularly rapid phases of decline were observed in the 1980’s in some subplots, and since 2000 in others, although the timing of rapid BA declines was not consistent across all subplots. Subplots whose BA declined by >25% initially had a basal area of approximately 49 m^2^ ha^-1^ in 1964 but this declined to 23 m^2^ ha^-1^ by 2014. The overall pattern of response, revealed by linear mixed models, was linear (slope = 0.52 ±0.15, p<0.001, R^2^ = 0.24). Similarly, a linear response of BA was observed in subplots whose BA declined by <25% or was stable, but with a positive trend overall (slope = 0.14±0.02, p<0.001, R^2^ = 0.03, Fig 1b).

### Tree recruitment

**Fig 2:**
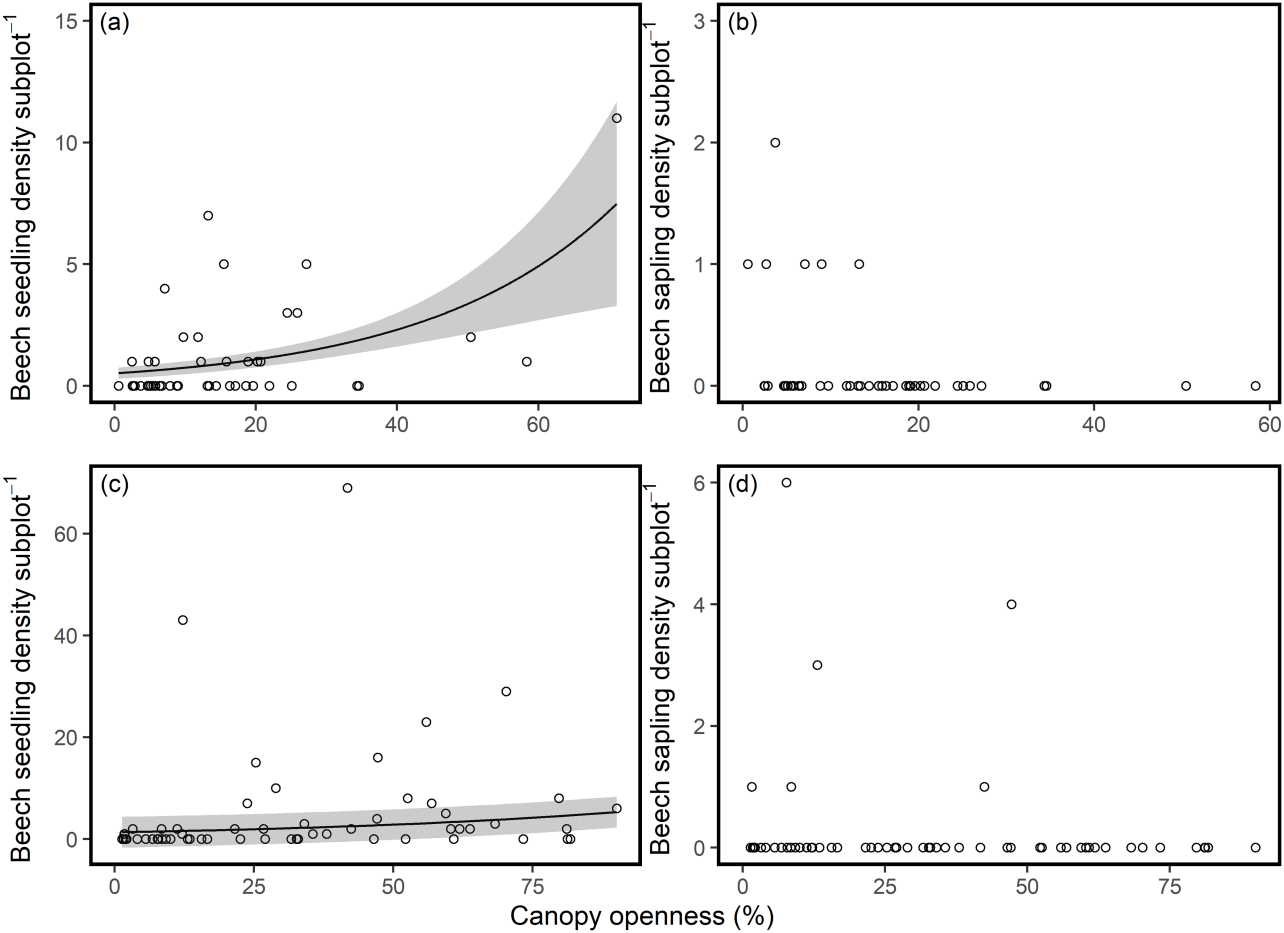
Relationships between juvenile tree density and canopy openness. Relationships between density of beech (a, c) seedlings and (b, d) saplings and canopy openness in woodlands in the New Forest showing signs of dieback. Graphs a and b use data from Denny Wood while graphs c and d use data collected from the gradient sites (12 sites). Solid lines represent predictions from coefficients with P ≤ 0.05 and grey bands represent 95% confidence intervals of these predictions.

Regression analyses indicated that in the Denny Wood data, canopy openness was positively related to beech seedling density (slope = 0.56 ± 0.09, P <0.001, R^2^ = 0.09, Fig 2a), with no other models having a ΔAICc≤7, indicating this as the best supported model. Similar relationships were observed for the gradient sites, with a positive relationship recorded between canopy openness and beech seedling density (slope = -0.41± 0.06, P <0.001, R^2^ = 0.16, Fig 2c). There was no clear relationship between sapling density and canopy openness at Denny Wood or at the gradient sites (Fig 2b & 2d), with null models almost as well supported as models that suggested a relationship with canopy openness. Model averaged coefficients of this relationship had P values > 0.05. As the number of beech saplings declined in Denny Wood during the years 1964–2014, so did the mortality rates of these saplings, from a maximum of 4.07% per year in 1964–1984 to 0.50% in 1996–2014 (Table 1). Conversely the proportion of saplings that became mature trees (>10 cm DBH) showed an increase over this time period (Table 1). However, it is important to note that only seven saplings were recruited over the 50 years prior to 2014 (Table 1).

**Table 1:** Summary of recruitment and mortality of beech saplings in Denny Wood from 1964 to 2014.

Census period	No. of saplings	No. of saplings recruited from seedlings	No. of saplings that died during census	No. of saplings that increased beyond DBH 10 cm	Annual rate of increase to >10 cm DBH	Annual mortality rate of saplings
1964–1984	179	3	101	25	0.75%	4.07%
1984–1988	56	1	6	11	5.32%	2.79%
1988–1996	40	2	6	13	4.79%	2.01%
1996–2014	23	1	2	14	5.08%	0.50%

### Sapling and mature tree mortality

**Fig 3:**
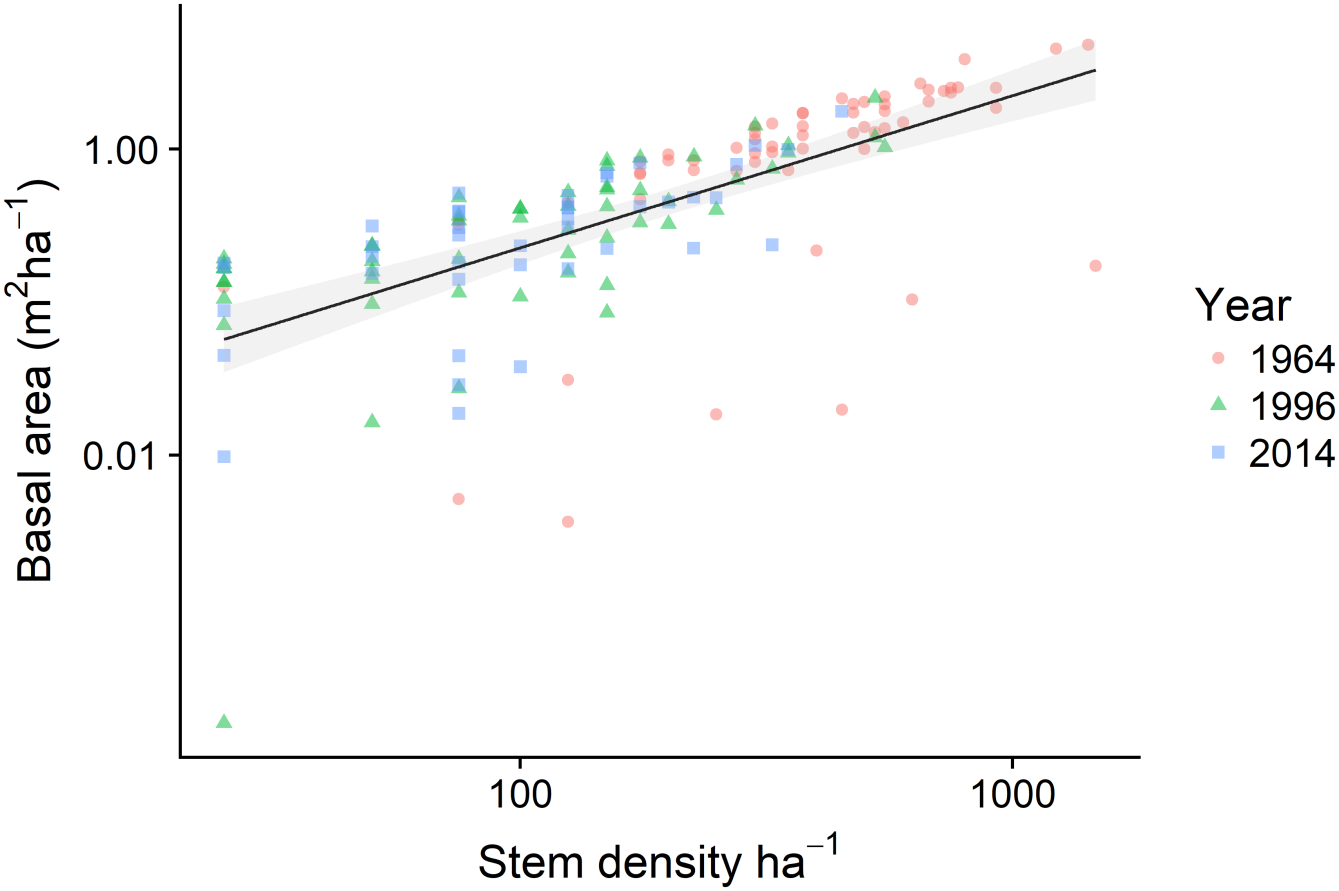
Relationship between subplot stem density and total subplot basal area. Points represent individual plots in 1964 (red circles), 1996 (green triangles) and 2014 (blue squares). The solid line represents the prediction from a mixed model of this relationship with the grey band representing the coefficient 95% confidence intervals. Note that both the x and y axes are log transformed.

The slope of the relationship between log subplot stem density and log subplot BA was positive (slope = 0.41 ± 0.05, marginal R^2^ = 0.24, Fig 3). However, in general subplots lost both stem density and BA between 1964 and 2014 (Fig 3). Given that self-thinning processes are strongest when plots are simultaneously increasing in biomass and losing stem density [46], such processes are unlikely to be responsible for the majority of tree death observed in Denny Wood from 1964–2014.

**Fig 4:**
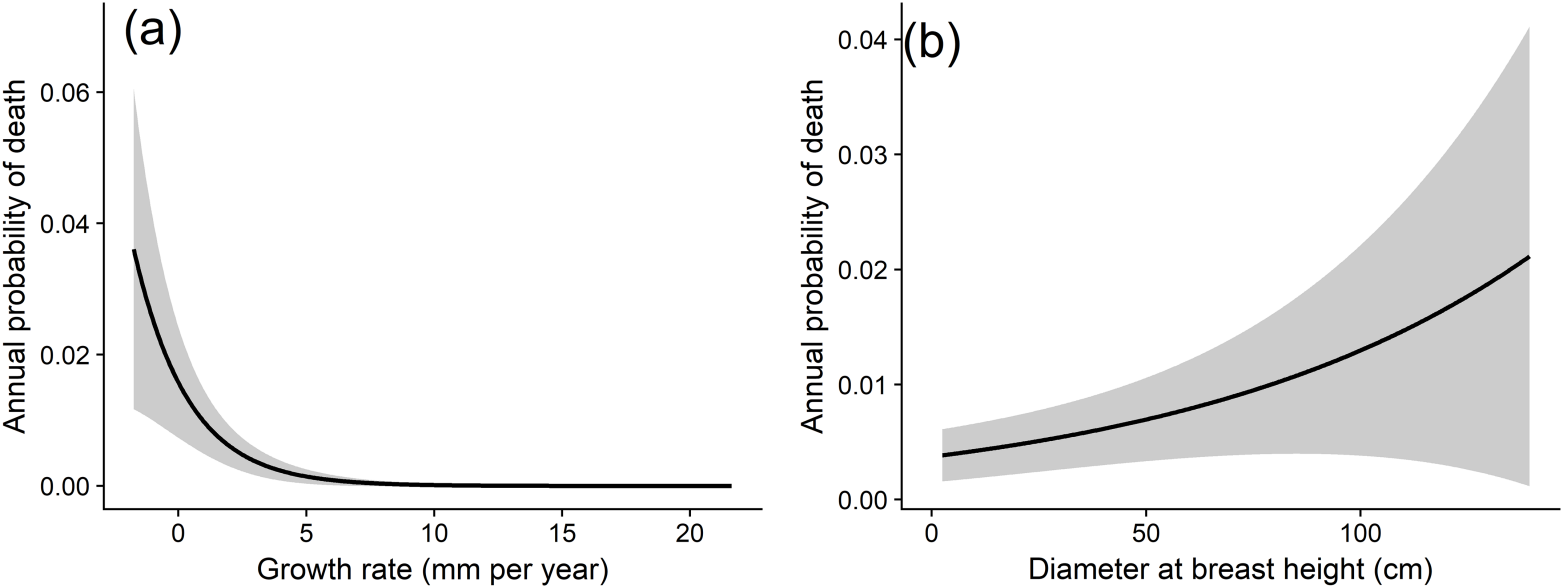
Predictors of annual beech mortality. Relationship between annual probability of beech tree death and (a) growth rate per year, (b) diameter at breast height (DBH). Lines represent predictions generated from model-averaged parameter estimates and grey bands represent 95% confidence intervals of predictions. Both growth rate per year and DBH were significant (P < 0.05) predictors of tree death. To produce predictions, all variables were held at their mean value apart from the variable of interest. For discussion of non-significant relationships, see results section.

When predicting the mortality of individual beech trees, growth rate was identified as the most important predictor, as it was included in all models with a ΔAICc≤7. Trees that grew slowly or not at all were more likely to die than those that grew relatively quickly (slope = -0.93 ± 0.15, P <0.001, Fig 4a). The next most important variable was DBH, with an importance value of 0.8. Models suggested that tree size was positively correlated with probability of mortality (slope = 0.23 ± 0.1, P = 0.045, Fig 4b). There was no significant relationship between distance to dead trees (slope = -0.11 ± 0.18, P = 0.46) or the clay content of soils and tree mortality (slope = -0.01 ± 0.07, P = 0.87). There was also no significant relationship between subplot stem density and tree mortality (slope = 0.05± 0.15, P = 0.73). When subjected to le Cessie-van Houwelingen-Copas-Hosmer goodness of fit tests, no model produced a P value ≤ 0.05, indicating that they all provide a reasonable fit to the data [38].

### Individual based model

**Fig 5:**
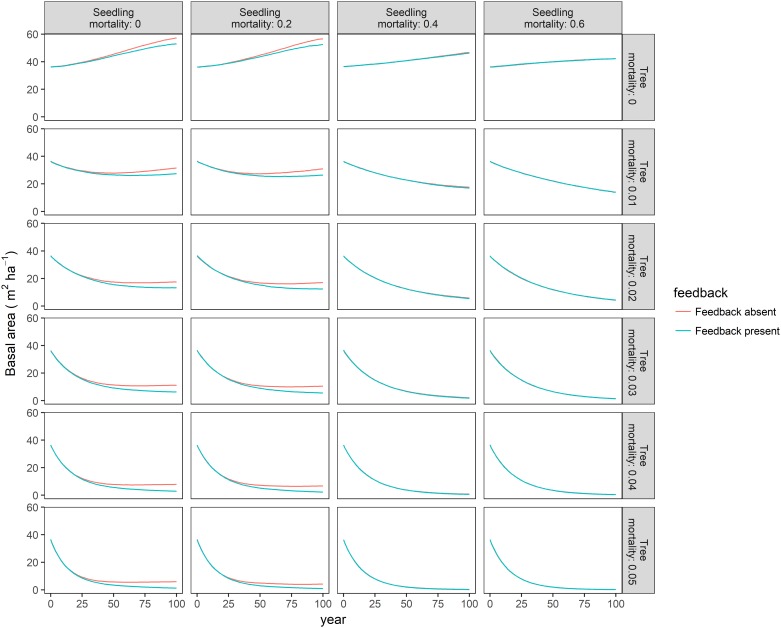
The effect of feedbacks in mature tree death and seedling mortality on predicted basal area over 100 years. Lines represent median basal area at each modelled time step, with red lines representing a model with no feedbacks and the blue lines representing a model with a spatial feedback in probability of death. Each graph represents a different combination of annual probability of juvenile mortality (columns) and mature mortality (rows), which are indicated numerically. Differences in seedling mortality are indicative of different intensities of herbivore pressure. For a more detailed version of this figure see S1 Fig.

Our individual based model indicated that critical values of seedling and sapling/mature tree mortality exist for the maintenance of forest structure. When annual sapling and mature tree mortality probability exceeded 0.01, BA declined, even when no seedlings died (Fig 5). When annual seedling tree mortality probability exceeded 0.4, recruitment of mature trees was close to zero and so BA declined (Fig 5, S3 Fig). Presence of a feedback in seedling mortality resulted in a more negative slope in BA when compared to the same scenario without feedbacks, especially when seedling and sapling/mature tree mortality were low (Fig 5). However, the effects of the feedback on BA were relatively slight overall. Regardless of whether or not the feedback was included in the simulations, none of the modelled scenarios suggested a convex curve indicative of a threshold response. The feedback in seedling mortality had a more pronounced negative effect on canopy cover (S2 Fig). Scenarios without feedbacks commonly had a canopy cover 10–20% higher than those with feedbacks. However, this was only true where seedling mortality was ≤0.4 –when seedling mortality was higher the trajectory of canopy cover was identical for scenarios with or without feedbacks in seedling mortality. Trends in stem density were similar to those seen in canopy cover (S3 Fig).

## Discussion

Our analysis indicates that basal area showed rapid, non-linear declines in a number of areas in a temperate forest, confirming that parts of it have undergone a dieback event that meet the criteria for an ecological threshold [2,12,47]. However, not all areas underwent these declines, suggesting that non-linear change has not occurred at the scale of the entire forest. This lack of transition was probably the result of heterogeneous timing and extent of mature tree mortality. Basal area decline was driven by low recruitment of beech combined with the death of large, old trees. Recruitment throughout the site was low and appears to be lower in forest gaps than in areas with high canopy cover, attributable to the high herbivore pressure. Slow growing, large trees showed increased risk of mortality, indicating ongoing disturbances [46]. However, the individual based model that we used to simulate forest dynamics at the site did not support our hypothesis that a positive feedback between canopy openness and herbivore pressure was greatly accelerating dieback.

Larger trees were more likely to die than smaller trees in Denny wood. This pattern is indicative of ongoing exogenous disturbances [46] and supports our hypothesis that mature tree mortality in the forest is related to drought and fungal pathogen attack by *Phytophthora spp*. [31]. However, the precise causes of tree death are difficult to determine. We could not find any evidence for the influence of soil structure or spatial contagion on patterns of mortality, which would have added more weight to these hypotheses, since *Phytophthora spp*. are thought to thrive in wet conditions that would be likely to occur in soils with higher clay content [48]. In addition we found no evidence that stem density influenced tree mortality as has been observed in some previous studies of drought affected forests [49–51]. These results highlight the difficulty of understanding the mortality processes of trees. For example, drought can increase tree mortality through the synergistic effects of insect damage [52], frost damage [53], fungal attack [48], or windthrow [54]. Spatial and temporal variation in such processes may explain the heterogeneity in mortality patterns that we observed in this study. Additional information on the mortality mode of the trees in our study might have helped to determine drivers of tree mortality [55].

We hypothesized that increased mortality of mature trees resulted in more areas with low canopy cover and that in these areas recruitment was limited because of a preference of ungulates to feed in forest gaps [56]. Over time we hypothesised that this could result in a more open forest and a transition to a non-forest state. However, we found limited evidence to support this hypothesis. There was a weak positive relationship between canopy openness and seedling density both in Denny Wood and in the gradient plots analysed, whereas the relationship between sapling abundance and canopy openness was less clear. This suggests that although initial seedling recruitment was positively related to canopy openness, seedling mortality was also higher in open areas of the forest, probably as a result of increased herbivore pressure. The strength of the relationship between seedling density and canopy openness may have been reduced for two reasons. Firstly, beech is a relatively shade tolerant species, and so any response to gaps may be reduced [57]. Secondly, herbivore abundance throughout the New Forest is high and so recruitment is likely to have been limited throughout Denny Wood [58], meaning that any increases in seedling numbers in gaps may have been short-lived.

When a feedback between seedling mortality as a result of increased grazing and canopy openness was included in the simulations for our individual based model, basal area declined more rapidly than comparable scenarios without this feedback. However, the difference was slight, and in no case was the feedback associated with an abrupt decline in basal area that would characterise a threshold response. This is because of the major influence of large, mature trees on basal area dynamics. Since the mortality of mature trees was unaffected by our proposed feedback, simulations did not result in rapid declines in forest structure. In addition, because herbivore pressure is high throughout the New Forest [33] we conclude that the relative impact of canopy openness on herbivore pressure, and consequently on regeneration at the forest scale, is likely to be slight. However, it is also clear that recruitment in Denny Wood is lower than in other sites in the New Forest [33]. Unfortunately, there are no quantitative data on the abundance of herbivores that would allow estimation of how recruitment relates to differences in herbivore pressure across the New Forest or at Denny Wood over time. However, given that our study shows that recruitment has been very low in Denny Wood since the 1960’s and previous work suggests that herbivore pressure has been high throughout this period [30,58], it appears that herbivory is the most likely cause of the recruitment failure we observed, as it is in many other temperate forests [59–61].

In summary, our results show evidence of a threshold response in basal area at the subplot scale, and the conversion of forest stands to grassland, which could be interpreted as an example of a tipping point leading to a regime shift [1,7]. This is likely to be driven by recruitment limitation as a result of herbivory and mature tree mortality resulting from drought and, possibly, a fungal pathogen. However, given that the shifts we observed did not occur at a stand scale it is unclear whether they should be interpreted as regime shifts. Although observation of large-scale, rapid regime shifts in forests is rare when they do occur it is often as a result of drivers that operate over large areas, that are severe enough to cause rapid tree death, and that result in positive feedbacks. These regime shifts often appear to depend on fire [10,11,62] or insect herbivores [63] as a primary driver of tree mortality.

Although it is unclear whether Denny Wood has undergone a regime shift, the results of our individual based model did suggest that there are critical values for mortality of saplings and mature trees that can lead to more rapid basal area decline, and avoidance of these should be considered a goal for future management. In addition, although we found little evidence of a rapid shift to a non-forest state, our study does suggest that reduced recruitment and death of large trees may result in a loss of tree cover over the next century. Reversing this decline should be seen as a priority for forest managers.

The vulnerability of large, old trees in our study as a result of drought and impaired recruitment has the potential to negatively impact biodiversity and ecosystem services. These trees provide key habitats for wildlife, as well as a range of ecosystem functions and services that are difficult to replace [64–66]. Recent papers (52–55) have identified that globally, large, old trees are threatened in many landscapes. High rates of herbivory have been highlighted as a particular problem in forested areas that are grazed by livestock, resulting in extremely low rates of recruitment [67–69]. Similar degradation of drought-prone forests has also been seen in the Western USA, where overgrazing by livestock and elk has limited regeneration [70]. Our findings add the New Forest to the list of locations where large, old trees are under threat.

In order to manage and conserve populations of large, old trees effectively policies and management practices should operate over much longer time scales than they do at present [65,71]. In the New Forest, the negative impacts of herbivores could be reduced by the fencing of forested areas [72]. Combining this with rotational grazing, where some forest areas would be allowed to regenerate before allowing grazing to resume, would allow traditional livestock grazing to be maintained in the forest whilst reducing problems associated with overgrazing [68]. Culling of deer and reduction or removal of livestock are also potential solutions to overgrazing, but the latter would be likely to be controversial in the New Forest, which has centuries old grazing rights [58]. Promoting regeneration may also help the forest to retain genetic diversity, thereby allowing greater resistance to more frequent and severe droughts that are forecast for the area. Under current management strategies native woodland cover will continue to decline, however if management is used to encourage regrowth and protect old trees, the native woodlands and their associated biodiversity in the New Forest can potentially recover from current drivers of degradation.

## Supporting information

S1 Fig The effect of feedbacks in mature tree death and juvenile mortality on predicted basal area over 100 years. Lines represent median basal area (BA) at each modelled time step, with red lines representing a model with no feedbacks and the blue lines representing a model with a spatial feedback in probability of death. Each graph represents a different combination of annual probability of juvenile mortality (columns) and mature mortality (rows), which are indicated numerically.
(PDF)

S2 Fig The effect of feedbacks in mature tree death and juvenile mortality on predicted canopy cover over 100 years. Lines represent median canopy cover at each modelled time step, with red lines representing a model with no feedbacks and the blue lines representing a model with a spatial feedback in probability of death. Each graph represents a different combination of annual probability of juvenile mortality (columns) and mature mortality (rows), which are indicated numerically.
(PDF)

S3 Fig The effect of feedbacks in mature tree death and juvenile mortality on predicted tree stem density over 100 years. Lines represent median stem density at each modelled time step, with red lines representing a model with no feedbacks and the blue lines representing a model with a spatial feedback in probability of death. Each graph represents a different combination of annual probability of juvenile mortality (columns) and mature mortality (rows), which are indicated numerically.
(PDF)

S1 File Detailed description of individual based model used in this study. (DOCX)

S2 File Individual based model used in this study. (NLOGO)

## References

[1] Scheffer M, Carpenter S, Foley JA, Folke C, Walker B. Catastrophic shifts in ecosystems. Nature. 2001;413: 591–6. doi: 10.1038/35098000 11595939 10.1038/35098000

[2] Groffman PM, Baron JS, Blett T, Gold AJ, Goodman I, Gunderson LH, et al Ecological Thresholds: The Key to Successful Environmental Management or an Important Concept with No Practical Application? Ecosystems. 2006;9: 1–13. doi: 10.1007/s10021-003-0142-z

[3] Brook BW, Ellis EC, Perring MP, Mackay AW, Blomqvist L. Does the terrestrial biosphere have planetary tipping points? Trends Ecol Evol. Elsevier Ltd; 2013;28: 396–401. doi: 10.1016/j.tree.2013.01.016 23453050 10.1016/j.tree.2013.01.016

[4] Barnosky AD, Hadly EA, Bascompte J, Berlow EL, Brown JH, Fortelius M, et al Approaching a state shift in Earth’s biosphere. Nature. Nature Publishing Group; 2012;486: 52–58. 10.1038/nature11018 22678279

[5] Lenton TM, Held H, Kriegler E, Hall JW, Lucht W, Rahmstorf S, et al Tipping elements in the Earth’s climate system. Proc Natl Acad Sci. National Acad Sciences; 2008;105: 1786–1793. 10.1073/pnas.0705414105 PMC2538841 18258748

[6] Andersen T, Carstensen J, Hernández-García E, Duarte CM, Hernandez-Garcia E, Duarte CM. Ecological thresholds and regime shifts: approaches to identification. Trends Ecol Evol. Elsevier; 2009;24: 49–57. doi: 10.1016/j.tree.2008.07.014 18952317 10.1016/j.tree.2008.07.014

[7] Briske DD, Washington-Allen RA, Johnson CR, Lockwood JA, Lockwood DR, Stringham TK, et al Catastrophic thresholds: a synthesis of concepts, perspectives, and applications. Ecol Soc. 2010;15: 37.

[8] Reyer CPO, Brouwers N, Rammig A, Brook BW, Epila J, Grant RF, et al Forest resilience and tipping points at different spatio-temporal scales: Approaches and challenges. J Ecol. 2015;103: 5–15. doi: 10.1111/1365-2745.12337

[9] Nepstad DC, Verissimo A, Alencar A, Nobre C, Lima E, Lefebvre P, et al Large-scale impoverishment of Amazonian forests by logging and fire. Nature. 1999;398: 505–508. doi: 10.1038/19066

[10] Barlow J, Peres CA. Fire-mediated dieback and compositional cascade in an Amazonian forest. Philos Trans R Soc Lond B Biol Sci. 2008;363: 1787–94. doi: 10.1098/rstb.2007.0013 18267911 10.1098/rstb.2007.0013 PMC2373873

[11] Acácio V, Holmgren M, Jansen PA, Schrotter O. Multiple Recruitment Limitation Causes Arrested Succession in Mediterranean Cork Oak Systems. Ecosystems. 2007;10: 1220–1230. doi: 10.1007/s10021-007-9089-9

[12] Huggett AJ. The concept and utility of “ecological thresholds” in biodiversity conservation. Biol Conserv. 2005;124: 301–310. doi: 10.1016/j.biocon.2005.01.037

[13] Thrush SF, Hewitt JE, Parkes S, Lohrer AM, Pilditch C, Woodin SA, et al Experimenting with ecosystem interaction networks in search of threshold potentials in real-world marine ecosystems. Ecology. Wiley Online Library; 2014;95: 1451–1457. 10.1890/13-1879.1 25039209

[14] Foley MM, Martone RG, Fox MD, Kappel C V, Mease LA, Erickson AL, et al Using Ecological Thresholds to Inform Resource Management: Current Options and Future Possibilities. Front Mar Sci. Frontiers; 2015;2: 95.

[15] Karr KA, Fujita R, Halpern BS, Kappel C V., Crowder L, Selkoe K a., et al Thresholds in Caribbean coral reefs: Implications for ecosystem-based fishery management. J Appl Ecol. Wiley Online Library; 2015;52: 402–412. doi: 10.1111/1365-2664.12388

[16] Filotas E, Parrott L, Burton PJ, Chazdon RL, Coates KD, Coll L, et al Viewing forests through the lens of complex systems science. Ecosphere. Wiley Online Library; 2014;5: 1–23.

[17] Lachat T, Bouget C, Bütler R, Müller J. 2.2 Deadwood: quantitative and qualitative requirements for the conservation of saproxylic biodiversity. Integr approaches as an Oppor Conserv For Biodivers. 2013; 92.

[18] Adams MA. Mega-fires, tipping points and ecosystem services: Managing forests and woodlands in an uncertain future. For Ecol Manage. Elsevier; 2013;294: 250–261. doi: 10.1016/j.foreco.2012.11.039

[19] Allen CD, Breshears DD, McDowell NG. On underestimation of global vulnerability to tree mortality and forest die-off from hotter drought in the Anthropocene. Ecosphere. Wiley Online Library; 2015;6: art129 doi: 10.1890/ES15-00203.1

[20] Teskey R, Wertin T, Bauweraerts I, Ameye M, McGuire MA, Steppe K. Responses of tree species to heat waves and extreme heat events. Plant Cell Environ. Wiley Online Library; 2015;38: 1699–1712. 10.1111/pce.12417 25065257

[21] Allen CD, Macalady AK, Chenchouni H, Bachelet D, McDowell N, Vennetier M, et al A global overview of drought and heat-induced tree mortality reveals emerging climate change risks for forests. For Ecol Manage. 2010;259: 660–684. doi: 10.1016/j.foreco.2009.09.001

[22] Millar CI, Stephenson NL. Temperate forest health in an era of emerging megadisturbance. Science (80-). 2015;349: 823–826. doi: 10.1126/science.aaa9933 26293954 10.1126/science.aaa9933

[23] Hirota M, Holmgren M, Van Nes EH, Scheffer M. Global resilience of tropical forest and savanna to critical transitions. Science (80-). 2011;334: 232–235. doi: 10.1126/science.1210657 21998390 10.1126/science.1210657

[24] Scheffer M, Hirota M, Holmgren M, Van Nes EH, Chapin FS. Thresholds for boreal biome transitions. Proc Natl Acad Sci U S A. 2012;109: 21384–9. doi: 10.1073/pnas.1219844110 23236159 10.1073/pnas.1219844110 PMC3535627

[25] Burrows MT, Schoeman DS, Buckley LB, Moore P, Poloczanska ES, Brander KM, et al The Pace of Shifting Climate in Marine and Terrestrial Ecosystems. Science (80-). 2011;334: 652–655. doi: 10.1126/science.1210288 22053045 10.1126/science.1210288

[26] Seidl R, Spies TA, Peterson DL, Stephens SL, Hicke J a. Searching for resilience: Addressing the impacts of changing disturbance regimes on forest ecosystem services. J Appl Ecol. 2016;53: 120–129. doi: 10.1111/1365-2664.12511 26966320 10.1111/1365-2664.12511 PMC4780065

[27] Cantarello E, Newton A, Martin P, Evans P, Gosal A, Lucash M. Quantifying resilience of multiple ecosystem services and biodiversity in a temperate forest landscape. Ecol Evol. 2017;0: 1–15. doi: 10.1002/ece3.3491 29187998 10.1002/ece3.3491 PMC5696413

[28] Scholes R, Settele J, Betts R, Bunn S, Leadley P, Nepstad D, et al Terrestrial and inland water systems In: Field C, Barros V, Mach K, Mastrandrea M, editors. Climate Change 2014: Impacts, Adaptation, and Vulnerability. Cambridge: Cambridge University Press; 2014 pp. 271–360.

[29] Mountford EP, Peterken GF, Edwards PJ, Manners JG. Long-term change in growth, mortality and regeneration of trees in Denny Wood, an old-growth wood-pasture in the New Forest (UK). Perspect Plant Ecol Evol Syst. 1999;2: 223–272. doi: 10.1078/1433-8319-00072

[30] Mountford EP, Peterken GF. Long-term change and implications for the management of wood-pastures: experience over 40 years from Denny Wood, New Forest. Forestry. 2003;76: 19–43. doi: 10.1093/forestry/76.1.19

[31] Martin PA, Newton AC, Cantarello E, Evans P. Stand dieback and collapse in a temperate forest and its impact on forest structure and biodiversity. For Ecol Manage. Elsevier B.V.; 2015;358: 130–138. doi: 10.1016/j.foreco.2015.08.033

[32] Evans PM, Newton AC, Cantarello E, Martin P, Sanderson N, Jones DL, et al Thresholds of biodiversity and ecosystem function in a forest ecosystem undergoing dieback. Sci Rep. 2017;7: 1–9. 28754979 10.1038/s41598-017-06082-6 PMC5533776

[33] Newton AC, Cantarello E, Appiah D, Perrella L, Newton AC, Lovegrove A, et al The influence of grazing animals on tree regeneration and woodland dynamics in the New Forest, England In: Rotherham I, editor. Trees, Forested Landscapes and Grazing Animals—A European Perspective on Woodlands and Grazed Treescapes. Routledge Oxford; 2013 pp. 163–179.

[34] Sheil D, Burslem DFRP, Alder D. The Interpretation and misinterpretation of mortality rate measures. J Ecol. 1995;83: 331–333. doi: 10.2307/2261571

[35] Westoby M. The self-thinning rule. Adv Ecol Res. 1984;14: 167–226.

[36] Weller DE. A Reevaluation of the -3 / 2 Power Rule of Plant Self-Thinning. 1987;57: 23–43.

[37] Fortin M, Bedard S, DeBlois J, Meunier S. Predicting individual tree mortality in northern hardwood stands under uneven-aged management in southern Québec, Canada. Ann For Sci. 2008;65: 205.

[38] le Cessie S, Van Houwelingen JC. A Goodness-of-Fit Test for Binary Regression Models, Based on Smoothing Methods. Biometrics. 1991;47: 1267–1282. doi: 10.2307/2532385

[39] Chao KJ, Phillips OL, Gloor E, Monteagudo A, Torres-Lezama A, Martínez RV, et al Growth and wood density predict tree mortality in Amazon forests. J Ecol. 2008;96: 281–292. doi: 10.1111/j.1365-2745.2007.01343.x

[40] Schielzeth H. Simple means to improve the interpretability of regression coefficients. Methods Ecol Evol. 2010;1: 103–113. doi: 10.1111/j.2041-210X.2010.00012.x

[41] Burnham KP, Anderson DR. Model selection and multimodel inference: a practical information-theoretic approach. Ecological Modelling. 2002 doi: 10.1016/j.ecolmodel.2003.11.004

[42] R Development Core Team. R: A Language and Environment for Statistical Computing. Vienna, Austria: R Foundation for Statistical Computing; 2011.

[43] Bates D, Maechler M, Bolker B, Walker S. lme4: Linear mixed-effects models using Eigen and S4. ArXiv. 2014;

[44] Barton K. MuMIn: Multi-model inference. R package. 2014.

[45] Wilensky U. Netlogo. Evanston, IL: enter for Connected Learning and Computer-Based Modeling, Northwestern University.; 1999.

[46] Coomes DA, Duncan RP, Allen RB, Truscott J. Disturbances prevent stem size-density distributions in natural forests from following scaling relationships. Ecol Lett. 2003;6: 980–989. doi: 10.1046/j.1461-0248.2003.00520.x

[47] Dodds WK, Clements WH, Gido K, Hilderbrand RH, King RS. Thresholds, breakpoints, and nonlinearity in freshwaters as related to management. J North Am Benthol Soc. BioOne; 2010;29: 988–997.

[48] Jung T. Beech decline in Central Europe driven by the interaction between Phytophthora infections and climatic extremes. For Pathol. 2009;39: 73–94. doi: 10.1111/j.1439-0329.2008.00566.x

[49] Bottero A, D’Amato AW, Palik BJ, Bradford JB, Fraver S, Battaglia MA, et al Density-dependent vulnerability of forest ecosystems to drought. J Appl Ecol. 2017; n/a—n/a. doi: 10.1111/1365-2664.12847

[50] Guarin A, Taylor AH. Drought triggered tree mortality in mixed conifer forests in Yosemite National Park, California, USA. For Ecol Manage. Elsevier; 2005;218: 229–244.

[51] Greenwood DL, Weisberg PJ. Density-dependent tree mortality in pinyon-juniper woodlands. For Ecol Manage. Elsevier; 2008;255: 2129–2137.

[52] Jactel H, Petit J, Desprez-Loustau M-L, Delzon S, Piou D, Battisti A, et al Drought effects on damage by forest insects and pathogens: a meta-analysis. Glob Chang Biol. Wiley Online Library; 2012;18: 267–276.

[53] Vanoni M, Bugmann H, Nötzli M, Bigler C. Drought and frost contribute to abrupt growth decreases before tree mortality in nine temperate tree species. For Ecol Manage. Elsevier B.V.; 2016;382: 51–63. doi: 10.1016/j.foreco.2016.10.001

[54] Bréda N, Huc R, Granier A, Dreyer E. Temperate forest trees and stands under severe drought: a review of ecophysiological responses, adaptation processes and long-term consequences. Ann For Sci. EDP Sciences; 2006;63: 625–644.

[55] Holzwarth F, Kahl A, Bauhus J, Wirth C. Many ways to die—partitioning tree mortality dynamics in a near-natural mixed deciduous forest. Zuidema P, editor. J Ecol. 2013;101: 220–230. doi: 10.1111/1365-2745.12015

[56] Kuijper DPJ, Cromsigt J, Churski M, Adam B, Jędrzejewski B, Jędrzejewski W. Do ungulates preferentially feed in forest gaps in European temperate forest? For Ecol Manage. Elsevier; 2009;258: 1528–1535.

[57] Packham JR, Thomas P a., Atkinson MD, Degen T. Biological Flora of the British Isles: Fagus sylvatica. J Ecol. 2012;100: 1557–1608. doi: 10.1111/j.1365-2745.2012.02017.x

[58] Newton A. Social-ecological resilience and biodiversity conservation in a 900-year-old protected area. Ecol Soc. The Resilience Alliance; 2011;16.

[59] Rooney TP. Deer impacts on forest ecosystems: a North American perspective. For An Int J For Res. Oxford University Press; 2001;74: 201–208.

[60] Gill RMA. A review of damage by mammals in north temperate forests: 3. Impact on trees and forests. For An Int J For Res. Oxford University Press; 1992;65: 363–388.

[61] Ammer C. Impact of ungulates on structure and dynamics of natural regeneration of mixed mountain forests in the Bavarian Alps. For Ecol Manage. Elsevier; 1996;88: 43–53.

[62] Airey Lauvaux C, Skinner CN, Taylor AH. High severity fire and mixed conifer forest-chaparral dynamics in the southern Cascade Range, USA. For Ecol Manage. 2016;363: 74–85. http://dx.doi.org/10.1016/j.foreco.2015.12.016

[63] van Mantgem PJ, Stephenson NL, Byrne JC, Daniels LD, Franklin JF, Fulé PZ, et al Widespread increase of tree mortality rates in the western United States. Science. 2009;323: 521–524. doi: 10.1126/science.1165000 19164752 10.1126/science.1165000

[64] Lindenmayer DDB, Laurance WFW, Franklin JJF. Global Decline in Large Old Trees. Science (80-). 2012;338: 1305–1306. doi: 10.1126/science.1231070 23224548 10.1126/science.1231070

[65] Lindenmayer DB, Laurance WF, Franklin JF, Likens GE, Banks SC, Blanchard W, et al New policies for old trees: averting a global crisis in a keystone ecological structure. Conserv Lett. 2013;0: n/a–n/a. doi: 10.1111/conl.12013

[66] Blicharska M, Mikusiński G. Incorporating social and cultural significance of large old trees in conservation policy. Conserv Biol. 2014;0: 1–10. doi: 10.1111/cobi.12341 25115905 10.1111/cobi.12341

[67] Gibbons P, Lindenmayer DB, Fischer J, Manning a D, Weinberg a, Seddon J, et al The future of scattered trees in agricultural landscapes. Conserv Biol. 2008;22: 1309–19. doi: 10.1111/j.1523-1739.2008.00997.x 18680500 10.1111/j.1523-1739.2008.00997.x

[68] Fischer J, Stott J, Zerger A, Warren G, Sherren K, Forrester RI. Reversing a tree regeneration crisis in an endangered ecoregion. Proc Natl Acad Sci. 2009;106: 10386–10391. doi: 10.1073/pnas.0900110106 19497886 10.1073/pnas.0900110106 PMC2691384

[69] Fischer J, Zerger A, Gibbons P, Stott J, Law BS. Tree decline and the future of Australian farmland biodiversity. Proc Natl Acad Sci U S A. 2010;107: 19597–602. doi: 10.1073/pnas.1008476107 20974946 10.1073/pnas.1008476107 PMC2984202

[70] Rogers PC, Mittanck CM. Herbivory strains resilience in drought-prone aspen landscapes of the western United States. J Veg Sci. 2014;25: 457–469. doi: 10.1111/jvs.12099

[71] Lindenmayer DB, Laurance WF. The Unique Challenges of Conserving Large Old Trees. Trends Ecol Evol. Elsevier Ltd; 2016;xx: 1–3. doi: 10.1016/j.tree.2016.03.003 27117523 10.1016/j.tree.2016.03.003

[72] Agra H, Schowanek S, Carmel Y, Smith RK, Ne’eman G. Forest Conservation In: Sutherland WJ, Dicks LV, Ockendon N, Smith RK, editors. What Works in Conservation 2017. Cambridge, UK: Open Book Publishers; 2017 pp. 285–328.

