# Supplementary figures and images for: Analysis of ecological thresholds in a temperate forest undergoing dieback
Source: PLoS One. 2017 Dec 14;12(12):e0189578. doi: 10.1371/journal.pone.0189578 (PMC5730120; doi:10.1371/journal.pone.0189578)

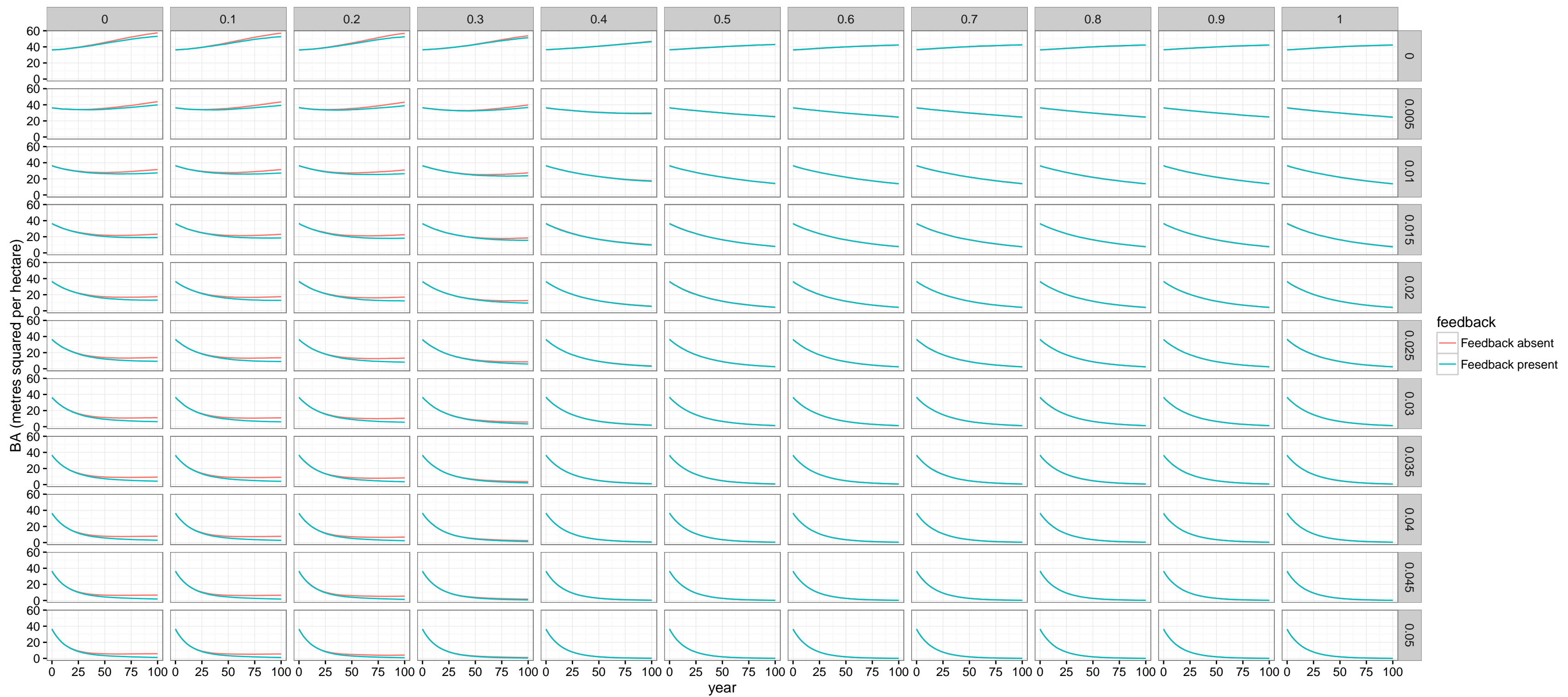

Supplement: S1 Fig — Lines represent median basal area (BA) at each modelled time step, with red lines representing a model with no feedbacks and the blue lines representing a model with a spatial feedback in probability of death. Each graph represents a different combination of annual probability of juvenile mortality (columns) and mature mortality (rows), which are indicated numerically. (PDF) [file pone.0189578.s001.pdf]

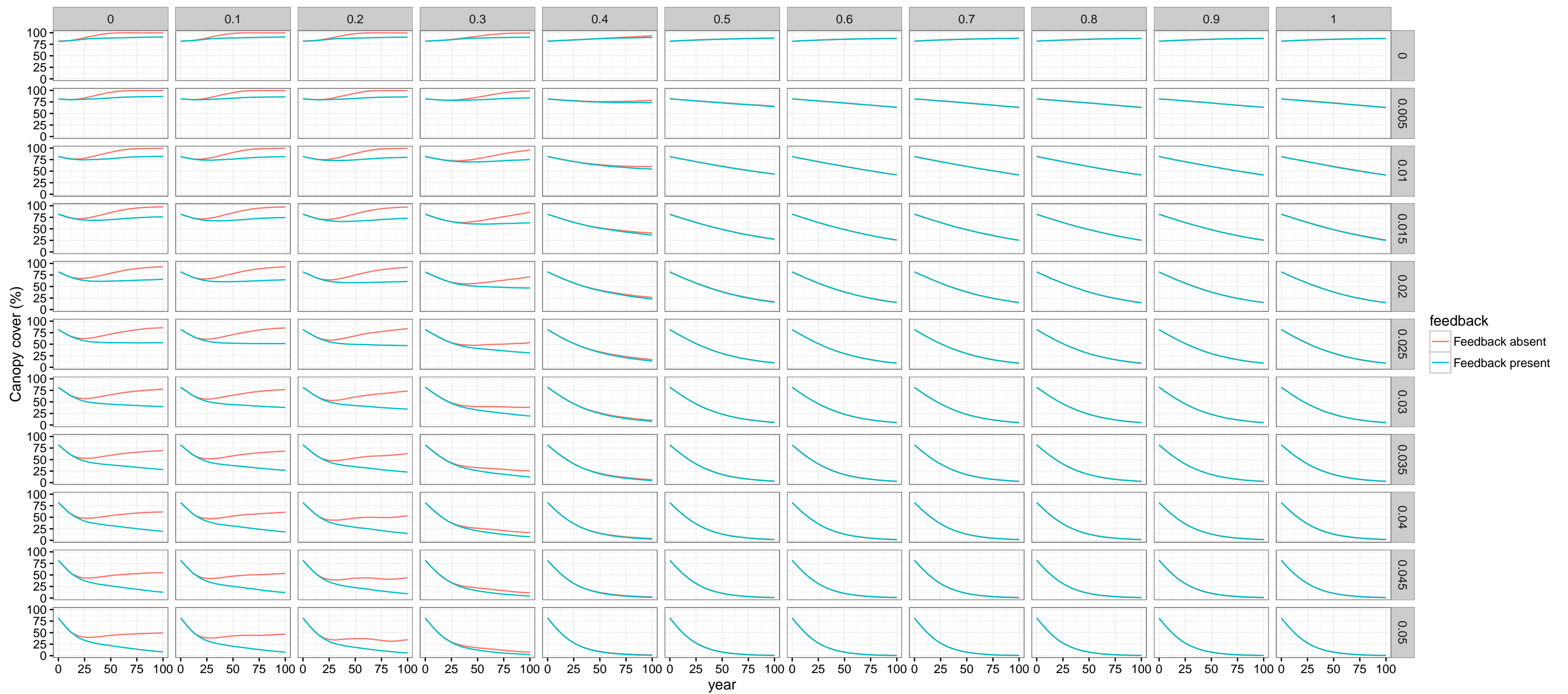

Supplement: S2 Fig — Lines represent median canopy cover at each modelled time step, with red lines representing a model with no feedbacks and the blue lines representing a model with a spatial feedback in probability of death. Each graph represents a different combination of annual probability of juvenile mortality (columns) and mature mortality (rows), which are indicated numerically. (PDF) [file pone.0189578.s002.pdf]

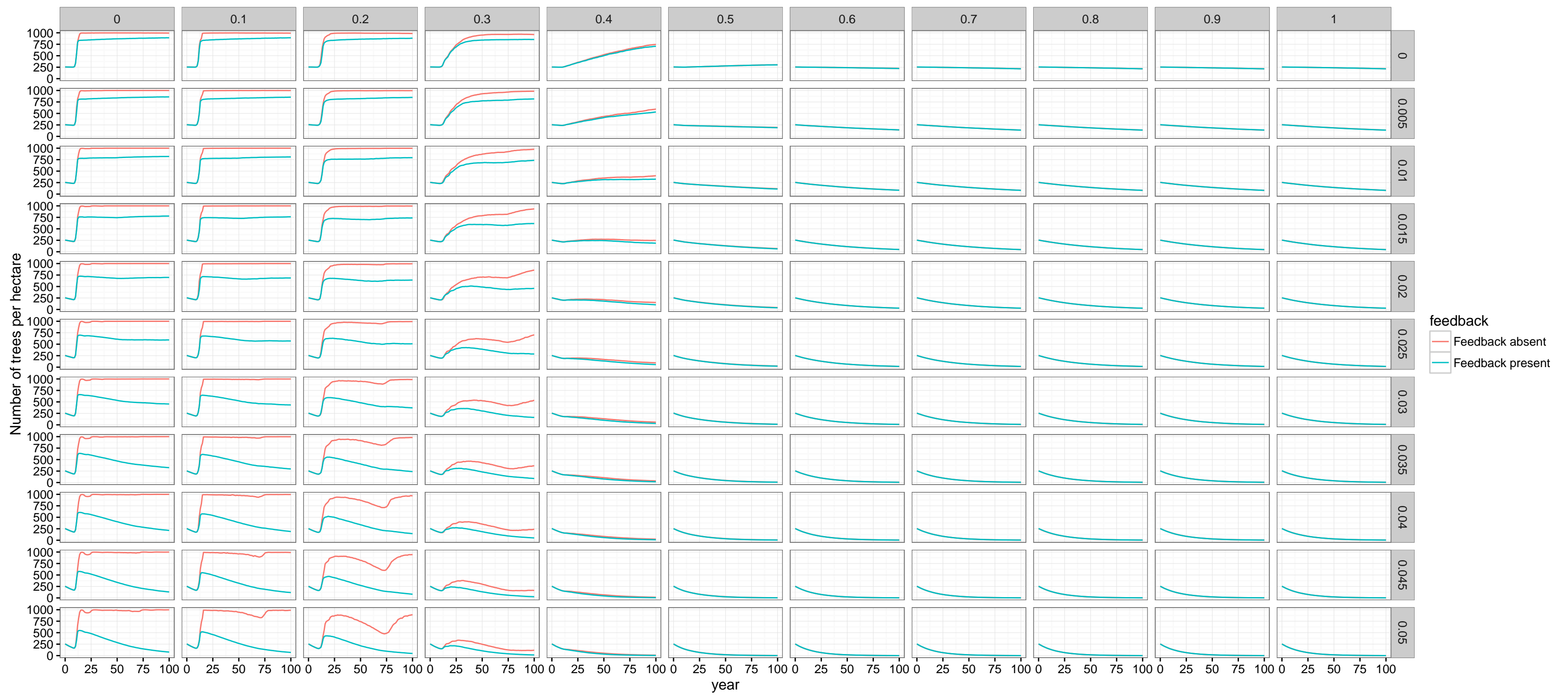

Supplement: S3 Fig — Lines represent median stem density at each modelled time step, with red lines representing a model with no feedbacks and the blue lines representing a model with a spatial feedback in probability of death. Each graph represents a different combination of annual probability of juvenile mortality (columns) and mature mortality (rows), which are indicated numerically. (PDF) [file pone.0189578.s003.pdf]
