# Supplementary material for: Analysis of ecological thresholds in a temperate forest undergoing dieback
Source: PLoS One. 2017 Dec 14;12(12):e0189578. doi: 10.1371/journal.pone.0189578 (PMC5730120; doi:10.1371/journal.pone.0189578)
Supplement: S1 File — (DOCX) [file pone.0189578.s004.docx]

**Supplementary materials**

**Description of Individual Based Model**

**Purpose**

We used our model to investigate under whether feedbacks in juvenile mortality could cause non-linear shifts in forest structure in a simplified representation of a New Forest beech woodland. The only species represented in the model is beech, as this is the dominant species found in the study area, and mortality of the species has caused the majority of BA loss in our study site, Denny Wood, from 1964-2014 [1].

**Entities, state variables and scales**

The model comprises of two types of entities: grid cells and individuals. Individuals represent seedlings or sapling/mature beech trees. Each seedling individual is characterised by its location, developmental stage, age (in years), and height (m). Each sapling/mature individual is characterised by its location, age (in years), DBH (cm), basal area (m^2^), height (m) and its crown area (m^2^). Initial DBH of mature trees is derived from the age of trees using an equation for beech growth defined in [2] and BA defined as $\left( \frac{DBH}{200} \right)^{2}\times\pi$. Height is calculated using the allometric equation of [3]:

$$H=1.3+\frac{{DBH}^{2}}{2.07+0.507DBH+0.0215{DBH}^{2}}$$

where $H$ is the the height of an individual beech tree and $DBH$is the diameter of the tree at breast height. Tree crown radius is calculated using the equation of [4]:

$$Cr=(1.04185 + 0.075 DBH) (1-{exp}^{\left( -\frac{DBH}{5.7292} \right)^{1.3341}})$$

By approximating the shape of a tree crown as a circle its area is calculated as:

$$CrArea={Cr}^{2}\times\pi$$

Canopy cover in each cell is calculated by summing the crown area of all trees that fall within it.

The model landscape consists of 22 x 22 grid cells, with the cells at the edge of the landscape considered unsuitable for individuals. Each cell in the landscape represents 10 m^2^, thus the entire area suitable for individuals represents 4 ha. Each model time step represents one year. Each grid cell is characterised by its location, the basal area of trees within it, canopy openness, the number of juveniles present in the grid cell, a list of the individual mature and juvenile trees in the grid cell and whether the cell can be colonised by trees. Each grid cell may contain up to 100 juveniles, and have a BA equivalent to the maximum observed at Denny Wood (90 m^2^ ha^-1^). Canopy openness in each grid cell was calculated as the sum of $CrArea$ for all trees within the grid cell.

**Process overview and scheduling**

Initially the distribution of individuals is determined by randomly distributing 1440 mature individuals with a random age drawn from an exponential distribution with a mean age of 81 years assigned to each individual. This was approximately the density and age structure of Denny Wood when it was surveyed in 2014. Following this 460 seedlings are randomly distributed across the space with their age drawn randomly from an exponential distribution with a mean of 5 m based on estimates from [5]. This represents the state in Denny Wood in 2014 when there were 115.22 ± 32.14 seedlings ha^-1^. Initially, juvenile height is determined by multiplying their age by 0.095 following the observations of beech juvenile growth rate by [6]. In each time step the following events are processed in the given order: identification of whether the time step represents a mast year, increase age of individuals by one year, increase mature individual DBH & BA, increase in juvenile individual height, seed dispersal from mature trees > 50 years old and death.

**Design concepts**

The total number of saplings and mature trees and basal area *emerge* from changes in the probability of tree mortality that occur as they age and increase in size, as well as from changes in the mortality of seedlings. *Interactions* between individuals are the result of density dependant mortality processes, which show size asymmetry. For juveniles this is modelled by defining a maximum number of juveniles that can coexist in any cell as 100. When these numbers are exceeded the smallest seedling is killed. Similarly, for mature trees the local maximum BA of any cell was set at 90 m^2^ ha^-1^, the maximum observed for any plot during 1964-2014. When this maximum is exceeded the smallest sapling or mature tree in a cell is killed. Juvenile growth rate is determined by mature tree canopy cover such that when canopy openness is >50% vertical growth is 12.6 cm year^-1^, otherwise the growth rate is 10.9 cm year^-1^ following the measurements of [7]. We observed in the field seedling density was higher in gaps, but this was not true of saplings, suggesting higher juvenile mortality in gaps. To simulate this we created a switch to control whether 100% of seedlings in gaps with canopy openness >50% died. This represents a potential feedback between mature tree and seedling mortality.

*Stochasticity* is used in the model to define whether a given time step represents a mast year. Beech trees produce large amounts of seed once every 2-3 years in the UK [8] and thus we set a probability of 0.3 of each year being a mast year providing masting had not occurred in the previous year. In addition, the chance of masting in a year following a mast year was set to zero since it is very rare for simultaneous masting years to occur in the UK [8,9].

**References**

1. Martin PA, Newton AC, Cantarello E, Evans P. Stand dieback and collapse in a temperate forest and its impact on forest structure and biodiversity. For Ecol Manage. Elsevier B.V.; 2015;358: 130–138. doi:10.1016/j.foreco.2015.08.033

2. Holzwarth F, Kahl A, Bauhus J, Wirth C. Many ways to die - partitioning tree mortality dynamics in a near-natural mixed deciduous forest. Zuidema P, editor. J Ecol. 2013;101: 220–230. doi:10.1111/1365-2745.12015

3. Kindermann GE. Die Flächenanteile der Baumarten. na; 1998.

4. Nagel J, Albert M, Schmidt M. Das waldbauliche Prognose-und Entscheidungsmodell BWINPro 6.1. Forst und Holz. M. & H. SCHAPER GMBH & CO. KG; 2002;57: 486–492.

5. Hasenkamp N, Ziegenhagen B, Mengel C, Schulze L, Schmitt HP, Liepelt S. Towards a DNA marker assisted seed source identification: A pilot study in European beech (Fagus sylvatica L.). Eur J For Res. 2011;130: 513–519. doi:10.1007/s10342-010-0439-3

6. Collet C, Lanter O, Pardos M. Effects of canopy opening on height and diameter growth in naturally regenerated beech seedlings. Ann For Sci. EDP Sciences; 2001;58: 127–134.

7. Ammer C, Stimm B, Mosandl R. Ontogenetic variation in the relative influence of light and belowground resources on European beech seedling growth. Tree Physiol. 2008;28: 721–728. doi:10.1093/treephys/28.5.721

8. Packham JR, Thomas P a., Atkinson MD, Degen T. Biological Flora of the British Isles: Fagus sylvatica. J Ecol. 2012;100: 1557–1608. doi:10.1111/j.1365-2745.2012.02017.x

9. Packham JR, Thomas PA, Lageard JGA, Hilton GM. the English Beech Masting Survey 1980–2007: Variation in the Fruiting of the Common Beech ( Fagus Sylvatica L.) and Its Effects on Woodland Ecosystems. Arboric J. 2008;31: 189–214. doi:10.1080/03071375.2008.9747535
